# Human whole genome genotype and transcriptome data for Alzheimer’s and other neurodegenerative diseases

**DOI:** 10.1038/sdata.2016.89

**Published:** 2016-10-11

**Authors:** Mariet Allen, Minerva M. Carrasquillo, Cory Funk, Benjamin D. Heavner, Fanggeng Zou, Curtis S. Younkin, Jeremy D. Burgess, High-Seng Chai, Julia Crook, James A. Eddy, Hongdong Li, Ben Logsdon, Mette A. Peters, Kristen K. Dang, Xue Wang, Daniel Serie, Chen Wang, Thuy Nguyen, Sarah Lincoln, Kimberly Malphrus, Gina Bisceglio, Ma Li, Todd E. Golde, Lara M. Mangravite, Yan Asmann, Nathan D. Price, Ronald C. Petersen, Neill R. Graff-Radford, Dennis W. Dickson, Steven G. Younkin, Nilüfer Ertekin-Taner

**Affiliations:** 1 Mayo Clinic, Department of Neuroscience, 4500 San Pablo Road, Jacksonville, Florida 32224, USA; 2 Institute for Systems Biology, 401 Terry Ave N., Seattle, Washington 98109, USA; 3 Mayo Clinic, Department of Health Sciences Research, 4500 San Pablo Road, Jacksonville, Florida 32224, USA; 4 Mayo Clinic, Department of Health Sciences Research, 200 First Street, Rochester, Minnesota 55905, USA; 5 Sage Bionetworks, 1100 Fairview Ave. N., Seattle, Washington 98109, USA; 6 University of Florida, Center for Translational Research in Neurodegenerative Diseases, 1275 Center Dr, Gainesville, Florida 32611, USA; 7 Mayo Clinic, Department of Neurology, 200 First Street, Rochester, Minnesota 55905, USA; 8 Mayo Clinic, Department of Neurology, 4500 San Pablo Road, Jacksonville, Florida 32224, USA

**Keywords:** Neurodegeneration, Genetics of the nervous system, Genome-wide association studies, RNA sequencing

## Abstract

Previous genome-wide association studies (GWAS), conducted by our group and others, have identified loci that harbor risk variants for neurodegenerative diseases, including Alzheimer's disease (AD). Human disease variants are enriched for polymorphisms that affect gene expression, including some that are known to associate with expression changes in the brain. Postulating that many variants confer risk to neurodegenerative disease via transcriptional regulatory mechanisms, we have analyzed gene expression levels in the brain tissue of subjects with AD and related diseases. Herein, we describe our collective datasets comprised of GWAS data from 2,099 subjects; microarray gene expression data from 773 brain samples, 186 of which also have RNAseq; and an independent cohort of 556 brain samples with RNAseq. We expect that these datasets, which are available to all qualified researchers, will enable investigators to explore and identify transcriptional mechanisms contributing to neurodegenerative diseases.

## Background & Summary

In the past decade GWAS identified risk loci for human diseases, including AD^1–7^ and other neurodegenerative diseases^8,9^. Despite this progress, a comprehensive understanding of the molecular mechanisms underlying these complex conditions remains elusive. This is partly due to the inability of the disease GWAS approach to identify the actual disease gene and the functional disease risk variants. We^10^ and others^11,12^ utilized combined gene expression GWAS (eGWAS) and disease GWAS to identify loci which harbor regulatory variants that confer disease risk and to nominate the actual disease genes at these loci. The underlying premise of these studies is that genetic variants that modulate expression levels of genes, which encode critical members of disease molecular pathways, will also influence disease risk^13^. If this is correct, then there should be significant overlap between disease GWAS and eGWAS variants, especially if assessed in the disease-relevant tissue. Indeed, in an eGWAS of brain tissue from subjects with AD and non-AD, comprised largely of other neurodegenerative diagnoses, we identified significant enrichment for disease GWAS variants for AD and other diseases^10^. We^14–18^ and others^8,19–22^ determined that many of the risk variants for AD and other neurodegenerative diseases influence brain levels of genes that are nearby in the genome. These studies implicate the genes that are likely to be involved in disease pathways, nominate regulatory variants as the functional disease risk factors and provide testable hypotheses for their downstream effects.

Most large-scale gene expression studies in human brains published to date^10,19,20,23^ utilize microarray-based gene or exon arrays. Despite the versatility, cost-effectiveness and large-scale utility, this approach has limitations, including restricted dynamic range, lack of probes for all known gene isoforms and confinement of assays to known transcripts. RNA sequencing (RNAseq) provides an attractive alternative that can surpass these limitations and provide much more in-depth information about the human transcriptome in a high-throughput manner^24^. To expand our prior work on the human transcriptome based on microarray approaches and to evaluate gene/exon/isoform levels in a comparative fashion between AD and other neurodegenerative diseases, we have generated RNAseq data on brain samples from both a subset of the subjects that underwent microarray transcriptome studies^18^ and also an independent cohort. These datasets will be of utility in performing expression quantitative trait loci (eQTL), expression profiling and network analyses to facilitate interpretation of genetic associations and further understanding of disease-mediated changes in transcriptional regulation.

The present report is a description of the large-scale human genetic, and both microarray- and RNAseq-based transcriptome datasets we generated. The datasets described in this report have been made available to the research community through the Accelerating Medicines Partnership in Alzheimer’s Disease (AMP-AD) Knowledge Portal (Data Citation 1). The portal is hosted in the Synapse software platform^25^ from Sage Bionetworks as part of a series of datasets developed in support of the AMP-AD Target Identification and Preclinical Validation Project. The AMP-AD consortium includes six academic teams that will be generating genomic data from human brain or blood samples collected from more than 10 cohorts. Datasets are hosted in a common environment with standardized meta-data and annotations to facilitate cross-cohort query, access, and analysis. Each dataset provides a unique perspective on AD; therefore, datasets differ in types, generation protocols, and underlying patient characteristics. Together, this collection represents to date the most comprehensive collection of human genomic data in the field and, as such, it will be invaluable to a broad set of researchers.

The datasets described herein include the following: (1) late-onset AD GWAS^1^ (Mayo LOAD GWAS) on 2,099 subjects (Data Citation 2); (2) Mayo eGWAS^10^ on 773 samples from the cerebellum (CER) and temporal cortex (TCX) brain regions from a subset of Mayo LOAD GWAS participants (Data Citations 3,4); (3) Mayo Pilot RNAseq^18^ generated on a subset of 186 TCX samples from the Mayo eGWAS (Data Citation 5); (4) Mayo RNAseq on an independent cohort of 556 TCX^26^ (Data Citation 6) and CER (Data Citation 7) samples from subjects with AD, progressive supranuclear palsy (PSP), pathologic aging and elderly controls without neurodegenerative diseases. This report provides a comprehensive understanding of these cohorts, a detailed description of subjects, samples, data generation, and quality control (QC) as well as instructions to access these rich datasets by the scientific community.

## Methods

**Figure 1:**
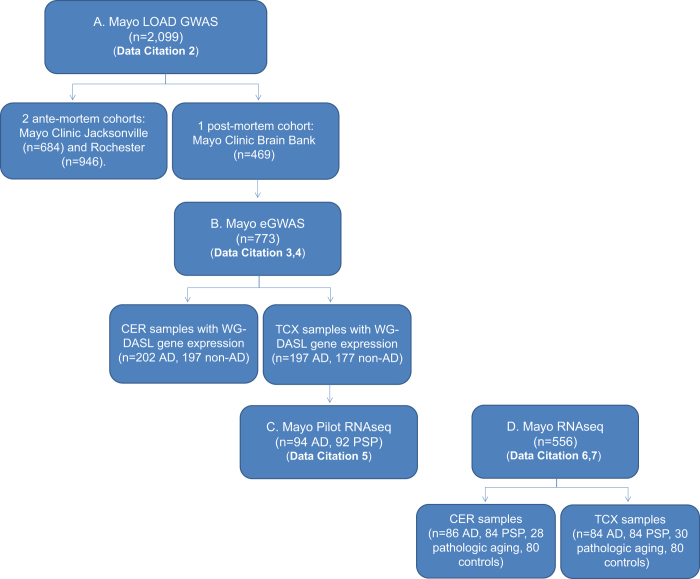
Overview of the relationship of the four genomic datasets herein described.

The repository of human whole genome genotype and transcriptome data described herein (Table 1,Fig. 1) consist of the following resources some of which have previously been published: Previously published datasets include whole genome genotype data from the **Mayo LOAD GWAS**^1^ (Data Citation 2) and microarray-based whole transcriptome data from the **Mayo eGWAS**^10^ (Data Citations 3,4). Next-generation RNA-sequencing (**RNAseq**) data from a subset of the patients from the Mayo Clinic eGWAS, referred to as the ‘**Mayo Pilot RNAseq**’ (Data Citation 5), was published in part^18^. A non-overlapping cohort with RNAseq-based transcriptome data named ‘**Mayo RNAseq**’ (Data Citations 6,7) has also been published in part^26^. For a comprehensive description of the overall repository, the data from the published studies are also described herein, albeit in an abbreviated fashion. These four study cohorts will be referred to by their names as mentioned above, preceded by letters A-D (Table 1) henceforth.

### Study Populations

All of this work was approved by the Mayo Clinic Institutional Review Board. All human subjects or their next of kin provided informed consent. The characteristics of the four study populations are as follows:

#### Mayo LOAD GWAS

The characteristics of the cohort for this study (Data Citation 2) were previously described in detail^1^. Briefly, this is a LOAD case versus control study composed in total of 2,099 subjects sourced from three different series, namely: Mayo Clinic Jacksonville, Mayo Clinic Rochester and Mayo Clinic Brain Bank series. These series are respectively termed as JS, RS and AUT in the GWAS publication^1^ (Table 1). Subjects in the Mayo Clinic Jacksonville and Mayo Clinic Rochester series were diagnosed clinically. These series consisted of 353 LOAD cases versus 331 controls; and 245 LOAD cases versus 701 controls. The Mayo Clinic Brain Bank series is a post-mortem cohort that consists of 246 LOAD cases versus 223 controls. All subjects were North American Caucasians. All clinical LOAD subjects were diagnosed as probable or possible AD, according to NINCDS-ADRDA criteria^27^. All clinical controls had a clinical dementia rating score of 0. LOAD subjects in the Mayo Clinic Brain Bank series met neuropathologic criteria for definite AD and had a Braak score of ≥4.0 (ref. 28), while controls did not meet neuropathologic criteria for AD, and each had Braak score of ≤2.5, which is an intermediary level of neurofibrillary tangle pathology between Braak score of 2 and 3; but most controls had neuropathologies unrelated to AD, including vascular dementia, frontotemporal dementia, dementia with Lewy bodies, multi-system atrophy, amyotrophic lateral sclerosis, and progressive supranuclear palsy. Ages, *APOE ε4* genotype and sex distribution for the Mayo LOAD GWAS cohort are shown in Table 2. This study only included subjects with ages between 60 and 80 years, based on the assumption that much of the genetic risk for LOAD will be concentrated in this age group, especially given the age-dependent effects of the strongest AD risk variant apolipoprotein E ε4 (*APOE4*)^28^. Age for the clinically diagnosed LOAD cases is defined as age at first diagnosis of AD, since age at onset is not always available. Age at entry into the study is used for the clinically diagnosed controls. Age at death is utilized for the cases and controls in the postmortem Mayo Clinic Brain Bank series, given that for this cohort, age at clinical diagnosis/ evaluation is not always available. Illumina Hap300 microarray genotypes from the subjects in these three case-control series were utilized to conduct a GWAS of LOAD risk^1^.

#### Mayo eGWAS

This cohort was previously described in detail^10^. All subjects in the Mayo eGWAS (Data Citations 3,4) are a subset of the Mayo Clinic Brain Bank series from the Mayo LOAD GWAS (Data Citation 2) (Fig. 1). The Mayo eGWAS is a whole transcriptome expression study in which brain samples from two different regions were analyzed, namely cerebellum (CER), which is relatively spared in AD, and temporal cortex (TCX), which is typically one of the first regions to be affected with AD neuropathology^29^. Transcriptome measurements were obtained from TCX of 202 AD subjects and from CER of 197 AD (Table 1). This study also included subjects without AD neuropathology, which are referred to as non-AD, given that many of these subjects had other neuropathologies. There were 197 non-AD subjects with TCX transcriptome measurements with the following neuropathologic diagnoses: progressive supranuclear palsy (PSP, *n*=107); Lewy body disease (LBD, *n*=25); corticobasal degeneration (CBD, *n*=22); frontotemporal lobar degeneration (FTLD, *n*=16); multiple system atrophy (MSA, *n*=11), vascular dementia (VaD, *n*=6); other (*n*=10). There were 177 non-AD subjects with CER transcriptome measurements that had the following neuropathologies: PSP (*n*=98); LBD (*n*=23); CBD (*n*=22); FTLD (*n*=15); MSA (*n*=7); VaD (*n*=4); other (*n*=8). Eighty-five percent of the subjects in the TCX cohort overlapped with those in the CER cohort. Demographics for the Mayo eGWAS subjects and samples, including RNA quality as assessed by RNA Integrity Numbers (RIN) are shown in Table 2.

#### Mayo Pilot RNAseq

All subjects in the Mayo Pilot RNAseq study (Data Citation 5) are a subset of the Mayo eGWAS (Data Citations 3,4), and are therefore also participants of the Mayo Clinic Brain Bank series that was included in the Mayo LOAD GWAS (Data Citation 2) (Fig. 1). The diagnostic categories in the Mayo Pilot RNAseq consist of 94 subjects with AD neuropathology and 92 PSP subjects, previously described^18,26^. PSP is a primary tauopathy characterized neuropathologically by neurofibrillary tangles (NFT) and tau-positive glial lesions^29,30^; and often presents clinically as a parkinsonian disorder. All PSP subjects were diagnosed neuropathologically by a single neuropathologist (DWD). For this study, only TCX samples were assessed (Table 2).

#### Mayo RNAseq

The subjects from this cohort are non-overlapping with the cohorts described above. The Mayo RNAseq cohort was utilized to generate RNAseq-based whole transcriptome data from 278 TCX^26^ (Data Citation 6) and 278 CER (Data Citation 7) samples. Two hundred thirty-eight subjects had both CER and TCX RNAseq and the rest had either CER or TCX RNAseq measurements based on tissue availability. CER samples were from the following diagnostic categories: 86 AD, 84 PSP, 28 pathologic aging and 80 controls without neurodegenerative diagnoses. TCX samples had the following diagnostic groups: 84 AD, 84 PSP, 30 pathologic aging and 80 controls. Control subjects each had Braak^28^ NFT stage of 3.0 or less, CERAD^31^ neuritic and cortical plaque densities of 0 (none) or 1 (sparse) and lacked any of the following pathologic diagnoses: AD, Parkinson’s disease (PD), DLB, VaD, PSP, motor neuron disease (MND), CBD, Pick’s disease (PiD), Huntington’s disease (HD), FTLD, hippocampal sclerosis (HipScl) or dementia lacking distinctive histology (DLDH). Subjects with pathologic aging also lacked the above diagnoses and had Braak NFT stage of 3.0 or less, but had CERAD neuritic and cortical plaque densities of 2 or more. None of the pathologic aging subjects had a clinical diagnosis of dementia or mild cognitive impairment. Given the presence of amyloid plaques, but not tangles and the absence of dementia, pathologic aging is considered to be either a prodrome of AD or a condition, in which there is resistance to the development of NFT and/or dementia^32^.

Within the Mayo RNAseq cohort (Data Citations 6,7), all AD and PSP subjects were from the Mayo Clinic Brain Bank, and all pathologic aging subjects were obtained from the Banner Sun Health Institute. Thirty-four control CER and 31 control TCX samples were from the Mayo Clinic Brain Bank, and the remaining control tissue was from the Banner Sun Health Institute. All subjects were North American Caucasians. All but control subjects, had ages at death ≥60, and a more relaxed lower age cutoff of ≥50 was applied for normal controls to achieve sample sizes similar to that of AD and PSP subjects. No upper age limit was imposed on this cohort, however when subjects had ages at death of ≥90, their ages were recorded as ‘90_or_above’ and shown as ‘90’ in Table 2 to protect patient confidentiality.

Table 2 details the demographic characteristics of the Mayo RNAseq cohort (Data Citations 6,7). PSP subjects tended to be younger than the other diagnostic groups. As expected, there was a greater frequency of *APOE4* positive subjects in the AD group, followed by pathologic aging, then PSP and control subjects. AD and pathologic aging subjects had greater female sex frequency (57%), followed by controls (49%), then PSP subjects (39%). RIN for all samples were selected to be ≥5.0. Pathologic aging and control samples had slightly lower RINs than AD and PSP samples, due to limitations in availability of samples in these former diagnostic categories.

### Molecular Data

#### Sample collection and processing

For the Mayo LOAD GWAS (A) (Data Citation 2), DNA samples were collected and processed as previously described^1^. For the antemortem Mayo Clinic Jacksonville and Mayo Clinic Rochester series, whole blood samples were collected in 10 ml EDTA tubes followed by DNA extraction using AutoGenFlex STAR instrument (AutoGen), whereas cerebellar tissue was used for DNA extraction from the postmortem Mayo Clinic Brain Bank series using the Wizard Genomic DNA purification kit (Promega). Given limited amounts of DNA from samples in the Mayo Clinic Rochester series and Mayo Clinic Brain Bank series, whole genome amplification (WGA) was applied using the Illustra GenomiPhi V2 DNA Amplification Kit (GE Healthcare Bio-Sciences), in four 5 ml reactions that utilized 5–15 ng genomic DNA as a template. Subsequent to the pooling of these reaction products, WGA DNA was subjected to quality control (QC) using SNP genotyping as previously described.

RNA extraction methods for the Mayo eGWAS^10^ (B) (Data Citations 3,4) and Mayo Pilot RNAseq^18^ (C) (Data Citation 5) were previously described. Total RNA was extracted from frozen brain samples using the Ambion RNAqueous kit (Life Technologies, Grand Island, NY) according to the manufacturer’s instructions. Brain samples for the Mayo RNAseq (D) (Data Citations 6,7) study underwent RNA extractions via the Trizol/chloroform/ethanol method, followed by DNase and Cleanup of RNA using Qiagen RNeasy Mini Kit and Qiagen RNase -Free DNase Set. The quantity and quality of all RNA samples were determined by the Agilent 2100 Bioanalyzer using the Agilent RNA 6000 Nano Chip (Agilent Technologies, Santa Clara, CA). Samples had to have an RNA Integrity Number (RIN) ≥5.0 for inclusion in either study (Table 2).

#### Data generation

The genotype data for the Mayo LOAD GWAS (A) (Data Citation 2) was generated using HumanHap300-Duo Genotyping BeadChips^1^, which were processed with an Illumina BeadLab station at the Mayo Clinic Genotyping Shared Resource (currently Mayo Clinic Medical Genome Facility=MGF, Rochester, Minnesota) according to the manufacturer’s protocols. Two samples were genotyped per chip for 318,237 SNPs across the genome. Genotype calls were made using the auto-calling algorithm in Illumina’s BeadStudio 2.0 software.

For the Mayo eGWAS study (B) (Data Citations 3,4), transcript levels were measured using the Whole Genome DASL assay (Illumina, San Diego, CA) as previously described^10^. Probe annotations were done based on NCBI RefSeq, Build 36.2. The RNA samples were randomized across the chips and plates using a stratified approach to ensure balance with respect to diagnosis, age, gender, RIN and *APOE* genotype. Raw probe mRNA expression data were exported from GenomeStudio software (Illumina Inc.) and preprocessed for background correction, variance stabilizing transformation, quantile normalization and probe filtering using the lumi package of BioConductor^33^.

Samples for both Mayo Pilot RNAseq (C) (Data Citation 5) and Mayo RNAseq (D) (Data Citations 6,7) studies were randomized prior to transfer to the Mayo Clinic MGF Gene Expression Core for library preparation and then the Sequencing Core for RNA sequencing. Mayo Pilot RNAseq (C) (Data Citation 5) AD and PSP samples were randomized across flowcells, taking into account age at death, sex and RIN. These samples underwent library preparation and sequencing at different times and therefore should be considered as separate datasets. Likewise, Mayo RNAseq (D) of TCX^26^ and CER samples (Data Citations 6,7, respectively) underwent RNAseq at different times. These samples were randomized across flowcells, taking into account age at death, sex, RIN, Braak stage and diagnosis. The TruSeq RNA Sample Prep Kit (Illumina, San Diego, CA) was used for library preparation from all samples. The library concentration and size distribution was determined on an Agilent Bioanalyzer DNA 1000 chip. All samples were run in triplicates using barcoding (3 samples per flowcell lane). For Mayo Pilot RNAseq (C) (Data Citation 5) samples, 50 base-pair, paired-end sequencing was done, whereas Mayo RNAseq (D) (Data Citations 6,7) samples underwent 101 bp, paired-end sequencing.

#### Data Processing

Mayo LOAD GWAS (A) (Data Citation 2) genotypes from Illumina BeadStudio 2.0 software were utilized to generate lgen, map and fam files that were imported into PLINK^34^ and converted to binary ped (.bed) and map (.bim) files, which are deposited together with PLINK format fam and covariate files (DOI and descriptions for each these files are provided in Table 3 (available online only)).

The Mayo eGWAS WG-DASL microarray expression dataset from TCX and CER (B) includes covariates and probe expression levels (Data Citation 3), which are preprocessed as published^10^ and described above. The Mayo eGWAS ‘eSNP Results’ (Data Citation 4) are the eQTL results from the test of association between the Mayo LOAD GWAS (Data Citation 2) genotypes and the WG-DASL gene expression measures analyzed by multivariable linear regression using an additive model in PLINK^34^, as published previously^10^ (DOI and descriptions for each these files are provided in Table 3 (available online only)). These analysis used preprocessed probe transcript levels as traits, SNP minor allele dosage as the independent variable, and adjusted for the following covariates: *APOE* ε4 dosage (0, 1, 2), age at death, sex, PCR plate, RIN and adjusted RIN squared (RIN-RINmean)^2^. Analyses were limited to SNP-probe pairs that were in-*cis*, defined as +/−100 kb of the targeted gene according to NCBI Build 36. The ADs and non–ADs were analyzed both separately and jointly. The joint analyses included diagnosis as an additional covariate (AD=1, non–AD=0). Results of analyses for both the genotyped SNPs as well as genotypes imputed to HapMap2 reference are provided. HapMap2 imputations were done as described^10^. The eGWAS results were previously made available through the NIAGADS repository (https://www.niagads.org/datasets/ng00025).

The Mayo Pilot RNAseq^18^ (Data Citation 5), Mayo RNAseq TCX^26^ and CER data (Data Citations 6,7, respectively) were processed using the same analytic pipeline. Read alignments were done using the SNAPR software^35^, an RNA sequence aligner based on SNAP, using GRCh38 reference and Ensembl v77 gene models. Outputs include per-sample gene and transcript counts, which are merged into a single file per data type (gene or transcript) that contains data for all samples across all genes/transcripts (DOI and descriptions for each these files are provided in Table 3 (available online only)). Alignment with SNAPR starts with the creation of hash indices built from both a reference genome GRCh38 and transcriptome GRCh38.77. SNAPR filters fastq reads by Phred score (>80% of the read must have a Phred score >= 20) and simultaneously aligns each read (or read pair) to both the genome and transcriptome. The best alignment is written to a sorted BAM file with read counts simultaneously tabulated and written for each sample. Read counts are given by gene ID and transcript ID (two separate files). We have previously tested the read counts generated by SNAPR to the read counts generated by HT-Seq and found them to be very comparable.

Post-processing was also performed using the same pipeline for these three RNAseq datasets as follows: The individual read count files produced by SNAPR are merged into a single file using two scripts: merge_count_files.R and a dataset-specific read-count merge script. These scripts generate the corresponding _counts.txt.gz files. The merged count files are normalized with the normalize_readcounts.R script, which uses the edgeR implementation of the trimmed mean of M-values (TMM) normalization method to calculate counts per million (CPM). These normalized counts are saved for both gene and transcript levels (DOI and descriptions for each these files are provided in Table 3 (available online only)).

#### Code Availability

The R script called merge_count_files.R^36^ was used to merge the RNAseq read count files produced by SNAPR into a single file, and can be found at https://github.com/CoryFunk/AMP-AD-scripts/blob/master/combine_count_files.pl. Also, the R script used to normalize the merged RNAseq read counts, called normalize_readcounts.R^36^, can be found at https://github.com/CoryFunk/AMP-AD-scripts/blob/master/tmm_normalization.R.

## Data Records

Data available for studies A-D (, , , , , ; Table 3 (available online only)) consists of a set of files that contain genomic, genetic or covariate data for a defined set of samples; analytic results are also provided when available. Data files can be found in the Sage Bionetworks AMP-AD Knowledge Portal (Data Citation 1) in study specific folders (and subfolders). Users can identify and search for data files and data descriptions using the unique Synapse ID and corresponding DOI provided in Table 3 (available online only). Each sample within a study has a unique sample ID, this sample ID is consistent across all files within the study, and files in other studies where applicable. The relationship between studies and sample overlaps is illustrated in Fig. 1. The samples in study C (Data Citation 5) are a subset of the samples in study B (Data Citation 3) which are likewise a subset of the samples in study A (Data Citation 2); the samples in study D (Data Citations 6,7) are independent of those in studies A-C. The Usage Notes section describes the data accession conditions, and the steps for requesting access.

## Technical Validation

### Data QC

Mayo LOAD GWAS (A) (Data Citation 2) QC methods were previously published^1^. Briefly, using PLINK^34^, subjects with genotyping call rates of <90%, duplicate genotyping and/or sex-mismatches between recorded and deduced sex were eliminated from the dataset. All SNPs with genotyping call rates <90%, minor allele frequencies <0.01, and/or Hardy-Weinberg p values <0.001 were also eliminated. Prior to QC, 318,237 SNPs were genotyped in 2,465 subjects. The available data includes the 313,504 SNP genotypes from 2,099 subjects that passed these QC parameters.

The Mayo eGWAS^10^ (B) (Data Citations 3,4) data was generated as follows: We annotated probes for presence of genetic variants by comparing their positions according to NCBI RefSeq, Build 36.3 to those of all variants within dbSNP131 and identified the list of probes that have ≥1 variants within their sequence. We depict this information in the files for the Mayo eGWAS, ‘eSNP Results’ (Data Citation 4) (Table 3 (available online only)), by including ‘SNP-In-Probe’ column, which has ‘TRUE’ if the probe sequence harbors ≥1 SNP, and ‘FALSE’, otherwise. We also calculated for each probe within each analytic group, percent detection rate above background. Probes that are detected in >12.5%, >25%, >50% and >75% of the subjects in each analytic group are annotated by four separate columns within the ‘eSNP Results’ (Data Citation 4) from the eGWAS that included HapMap2 imputed genotypes, described below. The purpose of these annotation columns is to enable others the flexibility to impose cutoffs based on presence/absence of variants within probe sequence and/or probe detection rates while providing the full dataset for completeness. The Mayo eGWAS (Data Citation 3,Data Citation 4) also included replicate samples as described for QC and to estimate intraclass coefficients (ICC), which is the between-subject variance, as a percentage of the total variance in probe expression^10^. There were 4 AD and 4 non-AD temporal cortex samples that were measured in 5 replicates; and 10 AD and 5 non-AD cerebellar sample replicates across five plates. Universal human RNA (UHR) samples were also run on each PCR plate as part of QC. The expression phenotypes include results from only one of the replicate subjects selected randomly and exclude UHR results. It should be noted that 3 AD and 9 non-AD subjects for TCX, and 4 AD subjects for CER, do not have associated GWAS genotypes as they did not pass≥1 GWAS QC parameter described above.

For the Mayo Pilot RNAseq^18^ (C) (Data Citation 5) data principal components analysis (PCA) identified 2 outliers in the AD and 4 in the PSP cohort. The covariates for these subjects were set to missing (=NA) in the respective covariate files (DOI and descriptions for these files are provide in Table 3 (available online only)). Hence, although 96 AD and 96 PSP subjects underwent sequencing in the Mayo Pilot RNAseq study, 94 AD and 92 PSP subjects were retained for analyses. It should be noted that of these subjects, 1 AD and 7 PSP subjects lack GWAS data due to either having genotype counts <90% or failing sex checks. PCA identified no outliers in the Mayo RNAseq (D) of TCX^26^ samples (Data Citation 6) but 2 such subjects in the CER analyses (Data Citation 7). The covariate data in the relevant CER files for these two subjects were set to missing. We likewise assessed the RNASeq data for sex discrepancies based on Y chromosome gene expression and documented sex and identified 2 subjects with mis-matched sex for both TCX and CER, plus a third subject in the CER cohort. These were also set to missing in the covariate files. At the time of this publication, the Mayo RNAseq subjects did not have GWAS genotypes deposited on Synapse.

## Usage Notes

The data described herein is available for use by the research community and has been deposited in the AMP-AD Knowledge Portal (). Table 3 (available online only) provides a detailed description of the files deposited for the four studies, their specific Synapse identifiers (IDs), DOIs, the types of files and definitions of the column headers. These files (, , , , , ), and their assigned DOIs will be maintained in perpetuity in the AMP-AD Knowledge Portal (). Access to all of these files is enabled through the Sage Bionetworks, Synapse repository; and a subset of the files for the Mayo LOAD GWAS () and the Mayo eGWAS (, ) are also available via NIAGADS (www.niagads.org).

The AMP-AD Knowledge Portal hosts data derived from multiple cohorts that were generated as part of or used in support of the AMP-AD Target Identification and Preclinical Validation project (). The portal uses the Synapse software platform^25^ for backend support, providing users with both web-based and programmatic access to data files. All data files in the portal are annotated using a standard vocabulary to enable users to search for relevant content across the AMP-AD datasets using programmatic queries. Data is stored in a cloud based manner hosted by Amazon web services (AWS), which enables user to execute cloud-based compute. Detailed descriptions including data processing, QC metrics, and assay and cohort specific variables are provided for each file as applicable.

Access for the data described herein is controlled in a manner set forth by the institutional review board (IRB) at the Mayo Clinic. All data use terms include: (1) maintenance of data in a secure and confidential manner, (2) respect for the privacy of study participants, (3) citation of the data contributors in any publications resulting from data use, and (4) informing data contributors of resultant publications. Specific data use terms are provided for each dataset (, , , ) under the header ‘Terms of use’; users must register for a Synapse account and provide electronic agreement to these terms prior to accessing the study files. Access to the Mayo LOAD GWAS data (A) () requires a data use certificate (doi:10.7303/syn2954402.2). User approvals are managed by the Synapse Access and Compliance Team (ACT).

Data on the AMP-AD Knowledge Portal are annotated with a common dictionary of terms (doi:10.7303/syn5478487.2) to enable querying of the data using the Synapse analytical clients (R client: syn1834618, python client: syn1768504, command line client: syn2375225). Fields, their allowable values specific to the datasets described herein and the dictionary of annotations are shown in Table 3 (available online only). These annotations can be used to identify files of interest within the available datasets and to filter on any of the fields using the allowable values from the dictionary (an example is shown here: doi:10.7303/syn5585666.1).

## Additional Information

**How to cite this article**: Allen, M *et al.* Human whole genome genotype and transcriptome data for Alzheimer's and other neurodegenerative diseases. *Sci. Data* 3:160089 doi: 10.1038/sdata.2016.89 (2016).

## Supplementary Material



## Figures and Tables

**Table 1:** Meta-data for each of the four studies.

**Study Name**	**Brief Description**	**Study Cohort/Sample type**	**N**	**Cohort Characteristics**	**Datatype**	**Platform**	**Reference**
A. Mayo LOAD GWAS (Data Citation 2)	LOAD Case control GWAS. Uses samples from 3 cohorts: Total 2,099 subjects (Post-QC). This data is used to identify loci associated with LOAD risk.	Mayo Clinic Jacksonville (JS)/Antemortem	*N*=353 cases, 331 controls	Clinical: AD Cases and Controls, collected at Mayo Clinic Jacksonville. Age at first diagnosis of AD or age at study entry: 60–80.	LOAD GWAS Genotypes, demographics	Illumina Hap 300	Carrasquillo *et al.*^1^, Nature Genetics
		Mayo Clinic Rochester (RS)/Antemortem	*N*=245 cases, 701 controls	Clinical: AD Cases and Controls, collected at Mayo Clinic Rochester. Age at first diagnosis of AD or age at study entry: 60–80.			
		Mayo Clinic Brain Bank (AUT)/ Postmortem	*N*=246 cases, 223 controls	Post-mortem: AD Cases (Braak ≥4.0) and Other Pathologies (Braak ≤2.5). Age at death: 60–80.			
B. Mayo eGWAS (Data Citations 3,4)	WG-DASL gene expression measures for a subset of Mayo Brain Bank subjects that were included in the Mayo LOAD GWAS: RNA was isolated from two brain regions: TCX and CER. This data is utilized to identify loci associated with brain gene expression in subjects with AD, subjects with Other brain pathologies that do not meet criteria for AD (Non-AD), and the combined cohort.	Mayo Brain Bank/Temporal Cortex	*N*=202 AD, 197 Non-AD controls	Post-mortem: AD Cases (Braak ≥4.0) and Other Pathologies (Braak ≤2.5). Age at death: 60–80.	Gene expression phenotypes, eGWAS results, covariates	Illumina WG-DASL	Zou *et al.*^10^, PLoS Genetics
		Mayo Brain Bank/Cerebellum	*N*=197 AD, 177 Non-AD controls				
C. Mayo Pilot RNAseq (Data Citation 5)	RNAseq gene expression measures for a subset of Mayo Brain Bank subjects that were included in the Mayo LOAD GWAS: RNA was isolated from TCX. This data is utilized to identify loci associated with brain gene expression in subjects with AD and subjects with PSP.	Mayo Brain Bank/Temporal Cortex	*N*=94 AD, 92 PSP	Post-mortem: AD Cases (Braak ≥4.0) and pathologic diagnosis of PSP (Braak≤2.5). Age at death: 60–80.	Gene expression phenotypes, covariates	IlluminaHiSeq2000, 50 bp, paired end RNAseq	Allen *et al.*^18^, Neurology:Genetics
D. Mayo RNAseq (Data Citations 6,7)	RNAseq gene expression measures for subjects from the Mayo Brain Bank non-overlapping with the Mayo LOAD GWAS, and also from Banner Sun Health Institute. RNA was isolated from two brain regions: TCX and CER. This data is utilized to compare brain gene expression between different pairwise diagnostic groups.	Mayo Brain Bank and Banner Sun Health/Temporal Cortex	*N*=84 AD, 84 PSP, 30 pathologic aging, 80 controls	Post-mortem: AD Cases (Braak ≥4.0), pathologic diagnoses of PSP (Braak≤3), pathologic aging (Braak≤3) and elderly control brains (Braak≤3) without neurodegenerative diagnoses. Age at death≤60.	Gene expression phenotypes, covariates	IlluminaHiSeq2000, 101 bp, paired end RNAseq	NA
		Mayo Brain Bank and Banner Sun Health/Cerebellum	*N*=86 AD, 84 PSP, 28 pathologic aging, 80 controls				

**Table 2:** Demographics for the cohorts included in each of the four studies.

			**A. Mayo LOAD GWAS (Data Citation 2)**		**B. Mayo eGWAS (Data Citations 3,4)**		**C. Mayo Pilot RNAseq (Data Citation 5)**	
			**TCX**		**CER**		**TCX**	
**Variables**	**AD (***n*=**844)**	**CON (1,255)**	**AD (***n*=**202)**	**NON-AD (***n*=**197)**	**AD (***n*=**197)**	**NON-AD (***n*=**177)**	**AD (***n*=**94)**	**PSP (***N*=**92)**
**Mean Age**±**s.d. (Range)**	74.0±4.8 (60–80)	73.2±4.4 (60–80)	73.6±5.5 (60–80)	71.6±5.6 (60–80)	73.6±5.6 (60–80)	71.7±5.5 (60–80)	74.1±5.7 (60–80)	71.9±5.4 (60–80)
**APOE4 positive/negative/null (%APOE4 positive)**	549/277/18 (65%)	344/889/22 (27%)	123/79/0 (61%)	49/146/2 (25%)	126/71/0 (64%)	45/130/2 (25%)	58/36/0 (62%)	20/72/0 (22%)
**Female (%)**	482 (57%)	641 (51%)	108 (53%)	78 (40%)	101 (51%)	63 (36%)	41 (44%)	37 (40%)
**Mean RIN**±**s.d. (Range)**	NA	NA	6.3±0.9 (5–9)	6.9±1.0 (5–9.3)	7.2±1.0 (5–9.4)	7.2±1.0 (5–9)	7.0±0.7 (6.2–9)	7.0±0.9 (5.7–9.3)

	**D. Mayo RNAseq (Data Citations 6,7)**							
	**TCX**				**CER**			
**Variables**	**AD (***n*=**84)**	**PSP (***n*=**84)**	**Path Aging (***n*=**30)**	**Control (***n*=**80)**	**AD (***n*=**86)**	**PSP (***n*=**84)**	**Path Aging (***n*=**28)**	**Control (***n*=**80)**
**Mean Age**±**s.d. (Range)**	82.4±7.7 (60–90)	74.0±6.5 (61–89)	85.2±4.3 (76–90)	82.6±8.8 (53–90)	82.5±7.7 (60–90)	74.0±6.5 (61–89)	84.7±4.3 (76–90)	82.5±8.3 (58–90)
**APOE4 positive/negative (%APOE4 positive)**	43/41 (51%)	13/71 (15%)	10/20 (33%)	10/70 (13%)	43/43 (50%)	13/71 (15%)	9/19 (32%)	11/69 (14%)
**Female (%)**	48 (57%)	33 (39%)	17 (57%)	39 (49%)	49 (57%)	33 (39%)	16 (57%)	39 (49%)
**Mean RIN**±**s.d. (Range)**	8.6±0.5 (7.7–10.0)	8.5±0.5 (7.8–10.0)	7.4±1.0 (5.3–8.9)	7.6±1.0 (5.3–9.7)	8.3±0.8 (5.7–10.0)	8.4±0.9 (5.5–10.0)	7.5±1.0 (5.7–9.0)	7.6±1.0 (5.5–9.7)

**Table 3:** Description of data files deposited in the AMP-AD Knowledge Portal.

**Study Name**	**doi**	**Data Type**	**File Name**	**doi**	**File Type**	**File Column Headers Definitions**	**Reference**
A. Mayo LOAD GWAS (Data Citation 2)	10.7303/syn2910256	Genotype and Covariate	MayoLOADGWAS_SNPGenotypes_covariates.csv	10.7303/syn3205821.6	Covariate information for subjects included in Mayo LOAD GWAS, comma delimited text file	FID=Family ID; IID=Individual ID; RSGWAS=‘1’ indicates if sample is part of Mayo Clinic Rochester Antemortem Cohort; AUTGWAS=‘1’ indicates if sample is part of Mayo Clinic Brain Bank Postmortem Cohort; AgeOver60=number of years beyond the age of 60 for age at death (Mayo Clinic Brain Bank Postmortem Cohort) or age at diagnosis (Mayo Clinic Rochester and Jacksonville Antemortem Cohorts); Sex=1 if Male; APOE4_Dose (+/−)=‘1’ indicates carriers of at least one copy of the APOEε4 allele; APOE4_dosage (0,1,2)= number of APOEε4 alleles ; APOE_Genotype=APOE genotype calls. For all columns ‘−9’ indicates missing data.	Carrasquillo *et al.*, ^1^, Nature Genetics
			MayoLOADGWAS_SNPGenotypes.bed	10.7303/syn3205812.4	LOAD GWAS binary ped file (PLINK format), genotype information	Binary File, No Column Headers.	
			MayoLOADGWAS_SNPGenotypes.bim	10.7303/syn3205814.4	LOAD GWAS binary map file (PLINK format), variant information	Binary File, No Column Headers.	
			MayoLOADGWAS_SNPGenotypes.fam	10.7303/syn3205816.4	LOAD GWAS fam file (PLINK format), pedigree and phenotype information	Six columns: Column 1=Family ID; Column 2= Individual ID; Column 3=Paternal ID; Column 4=Maternal ID; Column 5=Sex (1=male; 2=female); Column 6=Phenotype (1=control; 2= AD case)	
B. Mayo eGWAS (Data Citation 3,Data Citation 4)	10.7303/syn3157225	Array Expression and Covariate (Data Citation 3)	MayoEGWAS_arrayExpression_CBE_covariates.csv	10.7303/syn3256502.1	Covariate information for subjects with cerebellum expression measures, comma delimited text file.	Dxn=Diagnosis (0=Non-AD, 1=AD); Sex=1 if female and 0 if male; Age=Age at Death; E4dose=Number of APOEε4 alleles; plate1-plate4 (cerebellum) or plate 2-plate 5 (temporal cortex)=PCR plate - technical covariate; RIN=RNA intergrity number - a numerical assessment of the integrity of RNA; RINsqAdj=(RIN-RINmean)^2 - a statistical adjustment of the RIN.	Zou *et al.*, ^10^, PLoS Genetics
			MayoEGWAS_arrayExpression_TCX_covariates.csv	10.7303/syn3617056.1	Covariate information for subjects with temporal cortex expression measures, comma delimited text file.		
			MayoEGWAS_arrayExpression_CBE.csv	10.7303/syn3256501.1	Gene expression phenotypes from cerebellum tissue samples, comma delimited text file.	FID=Family ID; IID=Individual ID; ILMN_1762337 to ILMN_2137536=WG-DASL Illumina Probe ID.	
			MayoEGWAS_arrayExpression_TCX.csv	10.7303/syn3617054.1	Gene expression phenotypes from temporal cortex tissue samples, comma delimited text file.		
	10.7303/syn3157249	eSNP Results (Data Citation 4)	MayoEGWAS_analysis_eQTL_CBE_results_AD.gz	10.7303/syn3207163.3	Brain expression GWAS (eGWAS) results obtained using Hap300 genotypes in the cerebellar samples of the AD subjects, text file with 444,372 rows.	CHR=Chromosome; SNP=SNP rs number; BP=Physical position (base-pair) according to NCBI Ref Seq, Build 36.2; A1=Tested Allele; TEST=The ‘additive’ SNP genotype test; NMISS=Number of non-missing individuals included in analysis; BETA=Regression coefficient. Based on the SNP minor allele using an additive model; STAT=Coefficient t-statistic; P=Asymptotic p-value for t-statistic (uncorrected); PROBE=WG-DASL Illumina Probe ID; txStart=Starting base pair for the RefSeq gene the brain level of which is tested for associations. (Based on Build 36.2); txEnd=Ending base pair for the RefSeq gene the brain level of which is tested for associations. (Based on Build 36.2); SYMBOL=Gene symbol for the RefSeq gene tested in the eGWAS; hasSNP=If the probe sequence harbors ≥1 SNPs, then this is shown as ‘TRUE’ under the ‘SNP-In-Probe’ column, and ‘FALSE’, otherwise.	
			MayoEGWAS_analysis_eQTL_CBE_results_nonAD.gz	10.7303/syn3207167.3	Brain expression GWAS (eGWAS) results obtained using Hap300 genotypes in the cerebellar samples of the non-AD subjects, text file with 443,171 rows		
			MayoEGWAS_analysis_eQTL_CBE_results.gz	10.7303/syn3207165.3	Brain expression GWAS (eGWAS) results obtained using Hap300 genotypes in the cerebellar samples of the combined AD and non-AD subjects, text file with 443,784 rows		
			MayoEGWAS_analysis_eQTL_TCX_results_AD.gz	10.7303/syn3207169.3	Brain expression GWAS (eGWAS) results obtained using Hap300 genotypes in the temporal cortex samples of the AD subjects, text file with 450,813 rows.		
			MayoEGWAS_analysis_eQTL_TCX_results_nonAD.gz	10.7303/syn3207175.3	Brain expression GWAS (eGWAS) results obtained using Hap300 genotypes in the temporal cortex samples of the non-AD subjects, text file with 440,065 rows.		
			MayoEGWAS_analysis_eQTL_TCX_results.gz	10.7303/syn3207173.3	Brain expression GWAS (eGWAS) results obtained using Hap300 genotypes in the temporal cortex samples of the combined AD and non-AD subjects, text file with 445,356 rows.		
			MayoEGWAS_analysis_eQTL_CBE_imputedResults_AD.gz	10.7303/syn3207177.3	Brain expression GWAS (eGWAS) results obtained using HapMap2 imputed genotypes in the cerebellar samples of the AD subjects, text file with 4,427,924 rows.	CHR=Chromosome; SNP=SNP rs number; BP=Physical position (base-pair) according to NCBI Ref Seq, Build 36.2; A1=Tested Allele; TEST=The ‘additive’ SNP genotype test; NMISS=Number of non-missing individuals included in analysis; BETA=Regression coefficient. Based on the SNP minor allele using an additive model; STAT=Coefficient t-statistic; P=Asymptotic p-value for t-statistic (uncorrected); PROBE=WG-DASL Illumina Probe ID; txStart=Starting base pair for the RefSeq gene the brain level of which is tested for associations. (Based on Build 36.2); txEnd=Ending base pair for the RefSeq gene the brain level of which is tested for associations. (Based on Build 36.2); SYMBOL=Gene symbol for the RefSeq gene tested in the eGWAS; hasSNP=If the probe sequence harbors ≥1 SNPs, then this is shown as ‘TRUE’ under the ‘SNP-In-Probe’ column, and ‘FALSE’, otherwise. We also calculated for each probe within each analytic group, percent detection rate above background. Probes that are detected in >12.5%, >25%, >50% and >75% of the subjects in each analytic group are annotated by four separate columns labeled respectively and indicated with ‘TRUE’ and ‘FALSE’ statetments.	
			MayoEGWAS_analysis_eQTL_CBE_imputedResults_nonAD.gz	10.7303/syn3207181.3	Brain expression GWAS (eGWAS) results obtained using HapMap2 imputed genotypes in the cerebellar samples of the non-AD subjects, text file with 4,419,055 rows		
			MayoEGWAS_analysis_eQTL_CBE_imputedResults.gz	10.7303/syn3207179.3	Brain expression GWAS (eGWAS) results obtained using HapMap2 imputed genotypes in the cerebellar samples of the combined AD and non-AD subjects, text file with 4,483,512 rows		
			MayoEGWAS_analysis_eQTL_TCX_imputedResults_AD.gz	10.7303/syn3207183.3	Brain expression GWAS (eGWAS) results obtained using HapMap2 imputed genotypes in the temporal cortex samples of the AD subjects, text file with 4,426,363 rows.		
			MayoEGWAS_analysis_eQTL_TCX_imputedResults_nonAD.gz	10.7303/syn3207187.3	Brain expression GWAS (eGWAS) results obtained using HapMap2 imputed genotypes in the temporal cortex samples of the non-AD subjects, text file with 4,425,955 rows.		
			MayoEGWAS_analysis_eQTL_TCX_imputedResults.gz	10.7303/syn3207185.3	Brain expression GWAS (eGWAS) results obtained using HapMap2 imputed genotypes in the temporal cortex samples of the combined AD and non-AD subjects, text file with 4,484,344 rows.		
C. Mayo Pilot RNAseq (Data Citation 5)	10.7303/syn3157268	RNA-seq Alignment, Expression and Covariate	MayoPilotRNAseq Alzheimers Disease RNAseq BAMs	10.7303/syn5580964	Folder of BAM alignment files, 1 file per sample. Sample ID (IID) for each subject is provided in the first 12 digits of each file name.	NA	Allen *et al.*, ^18^, Neurology:Genetics
			MayoPilotRNAseq Progressive Supranuclear Palsy RNAseq BAMs	10.7303/syn5584594	Folder of BAM alignment files, 1 file per sample. Sample ID (IID) for each subject is provided in the first 12 digits of each file name.		
			MayoPilotRNAseq_RNAseq_TCX_AD_covariates.csv	10.7303/syn3607480.2	Covariate information for AD subjects with temporal cortex expression measures, comma delimited text file.	IlluminaSampleID=Individual ID (matches corresponding IID in Mayo LOAD GWAS files); Sex=M if Male and F if Female; Age at death; RIN=RNA intergrity number - a numerical assessment of the integrity of RNA; RINsqAdj=(RIN-RINmean)^2 - a statistical adjustment of the RIN; Library Batch=RNAseq library preparation batch; FCC1MR9ACXX - FCD1LUUACXX (AD) or FCD1GH3ACXX -FCC1CDJACXX (PSP)=‘1’ indicates sample was on sequencing flowcell - technical covariate; Flowcell=sequencing flowcell name.	
			MayoPilotRNAseq_RNAseq_TCX_PSP_covariates.csv	10.7303/syn3607506.1	Covariate information for PSP subjects with temporal cortex expression measures, comma delimited text file.		
			MayoPilotRNAseq_RNAseq_TCX_AD_geneCounts.tsv	10.7303/syn3607482.2	Gene expression phenotypes from AD temporal cortex tissue samples, space delimited text file with 64,254 rows.	Column 1=Ensemble Gene IDs; 1823480105_B - 1796012340_B=Illumina Sample ID's (matches corresponding IID in Mayo LOAD GWAS files).	
			MayoPilotRNAseq_RNAseq_TCX_AD_geneCounts_normalized.tsv	10.7303/syn3607497.1	Normalized gene expression phenotypes from AD temporal cortex tissue samples, tab delimited text file with 64,254 rows		
			MayoPilotRNAseq_RNAseq_TCX_AD_transcriptCounts.tsv	10.7303/syn3607485.2	Transcript expression phenotypes from AD temporal cortex tissue samples, space delimited text file with 208,245 rows.	Column 1=Ensemble Transcript IDs; 1823480105_B - 1796012340_B=Illumina Sample ID's (matches corresponding IID in Mayo LOAD GWAS files).	
			MayoPilotRNAseq_RNAseq_TCX_AD_transcriptCounts_normalized.tsv	10.7303/syn3607502.1	Normalized transcript expression phenotypes from AD temporal cortex tissue samples, tab delimited text file with 208,245 rows.		
			MayoPilotRNAseq_RNAseq_TCX_PSP_geneCounts.tsv	10.7303/syn3607487.2	Gene expression phenotypes from PSP temporal cortex tissue samples, space delimited text file with 64,254 rows	Column 1=Ensemble Gene IDs; 1811024586_A - 1811024534_A=Illumina Sample ID's (matches corresponding IID in Mayo LOAD GWAS files).	
			MayoPilotRNAseq_RNAseq_TCX_PSP_geneCounts_normalized.tsv	10.7303/syn3607513.1	Normalized gene expression phenotypes from PSP temporal cortex tissue samples, tab delimited text file with 64,254 rows		
			MayoPilotRNAseq_RNAseq_TCX_PSP_transcriptCounts.tsv	10.7303/syn3607489.2	Transcript expression phenotypes from PSP temporal cortex tissue samples, space delimited text file with 208,245 rows.	Column 1=Ensemble Transcript IDs; 1811024586_A - 1811024534_A=Illumina Sample ID's (matches corresponding IID in Mayo LOAD GWAS files).	
			MayoPilotRNAseq_RNAseq_TCX_PSP_transcriptCounts_normalized.tsv	10.7303/syn3607519.1	Normalized transcript expression phenotypes from PSP temporal cortex tissue samples, tab delimited text file with 208,245 rows.		
D. Mayo RNAseq (Data Citation 6,Data Citation 7)	10.7303/syn3163039	TCX RNA-seq Alignment, Expression and Covariate (Data Citation 6)	MayoRNAseq Temporal Cortex BAMs	10.7303/syn4894912	Folder of BAM alignment files, 1 file per sample. Sample ID (IID) for each subject is provided in the first 6 to 8 digits of each file name.	NA	NA
			MayoRNAseq_RNAseq_TCX_covariates.csv	10.7303/syn3817650.5	Covariate information for subjects with temporal cortex expression measures, comma delimited text file.	Sample ID=3 to 5 digit unique sample identifier followed by _TCX; Source=Pathology lab that provided the tissue; Tissue=Brain region sampled; RIN=RNA intergrity number - a numerical assessment of the integrity of RNA; Diagnosis=Pathological diagnosis at time of death (AD=Alzheimer's Disease; PSP=Progressive Supranuclear Palsy; Pathologic Aging; Control); Sex=M if Male, F if Female; AgeAtDeath; Flowcell=sequencing flowcell.	
			MayoRNAseq_RNAseq_TCX_geneCounts.tsv	10.7303/syn4650257.8	Gene expression phenotypes, temporal cortex tissue samples, space delimited text file with 64,254 rows	Column 1=Ensemble Gene IDs; 11344_TCX to 1047_TCX etc.=Sample ID's (matches corresponding SampleID in Covariate file).	
			MayoRNAseq_RNAseq_TCX_geneCounts_normalized.tsv	10.7303/syn4650265.4	Normalized gene expression phenotypes, temporal cortex tissue samples, tab delimited text file with 64,254 rows		
			MayoRNAseq_RNAseq_TCX_transcriptCounts.tsv	10.7303/syn5600752.2	Transcript expression phenotypes, temporal cortex tissue samples, space delimited text file with 208,245 rows	Column 1=Ensemble Transcript IDs; 11344_TCX to 1047_TCX etc.=Sample ID's (matches corresponding SampleID in Covariate file).	
			MayoRNAseq_RNAseq_TCX_transcriptCounts_normalized.tsv	10.7303/syn5600755.2	Normalized Transcript expression phenotypes, temporal cortex tissue samples, tab delimited text file with 208,245 rows		
	10.7303/syn5049298	CER RNA-seq Alignment, Expression and Covariate (Data Citation 7)	MayoRNAseq Cerebellum BAMs	10.7303/syn5049322	Folder of BAM alignment files, 1 file per sample. Sample ID for each subject is provided in the first 6 to 8 digits of each file name.	NA	
		MayoRNAseq_RNAseq_CBE_covariates.csv	10.7303/syn5223705.2	Covariate information for subjects with cerebellum expression measures, comma delimited text file.	Sample ID=3 to 5 digit unique sample identifier followed by _CER; Source=Pathology lab that provided the tissue; Tissue=Brain region sampled; RIN=RNA intergrity number - a numerical assessment of the integrity of RNA; Diagnosis=Pathological diagnosis at time of death (AD=Alzheimer's Disease; PSP=Progressive Supranuclear Palsy; Pathologic Aging; Control); Sex=M if Male, F if Female; AgeAtDeath; ApoE=APOE genotype; Flowcell=sequencing flowcell.		
		MayoRNAseq_RNAseq_CBE_geneCounts.tsv	10.7303/syn5201012.4	Gene expression phenotypes, cerebellum tissue samples, tab delimited text file with 64,254 rows	Column 1=Ensemble Gene IDs; 1000_CER to 991_CER= Sample ID's (matches corresponding SampleID in Covariate file).		
		MayoRNAseq_RNAseq_CBE_geneCounts_normalized.tsv	10.7303/syn5201007.4	Normalized gene expression phenotypes, cerebellum tissue samples, tab delimited text file with 64,254 rows			
		MayoRNAseq_RNAseq_CBE_transcriptCounts.tsv	10.7303/syn5600773.2	Transcript expression phenotypes, cerebellum tissue samples, space delimited text file with 208,245 rows	Column 1=Ensemble Transcript IDs; 1000_CER to 991_CER=Sample ID's (matches corresponding SampleID in Covariate file).		
		MayoRNAseq_RNAseq_CBE_transcriptCounts_normalized.tsv	10.7303/syn5600772.2	Normalized Transcript expression phenotypes, cerebellum tissue samples, tab delimited text file with 208,245 rows			
The Genotype and Covariate data from the Mayo LOAD GWAS study (A) and the eSNP results under the Mayo eGWAS study (B) were previously made available through the NIAGADS respository: https://www.niagads.org/datasets/ng00043 and https://www.niagads.org/datasets/ng00025 respectively.							
